# Hypersensitivity Reactions to Iodinated Contrast Media: A Narrative Review of Current Evidence and Clinical Challenges

**DOI:** 10.3390/healthcare13111308

**Published:** 2025-05-30

**Authors:** Francesca Losa, Giovanni Paoletti, Linda Borgonovo, Federica Buta, Stefania Merli, Serena Nannipieri, Marta Piantanida, Carlo Maria Rossi, Giada Sambugaro, Mona-Rita Yacoub, Vincenzo Patella, Giorgio Walter Canonica, Enrico Heffler, Maria Teresa Costantino

**Affiliations:** 1 SC Allergologia, Reumatologia e Immunologia Clinica, ASST Mantova, 46100 Mantova, Italy; francesca.losa@asst-mantova.it (F.L.); mariateresa.costantino@asst-mantova.it (M.T.C.); 2 Department of Biomedical Science, Humanitas University, 20072 Milan, Italy; giorgio_walter.canonica@hunimed.eu (G.W.C.); enrico.heffler@hunimed.eu (E.H.); 3 Personalized Medicine Asthma and Allergy Unit, IRCCS Humanitas Research Hospital, 20089 Milan, Italy; 4 SC Allergologia e Immunologia Clinica, ASST GOM Niguarda, 20162 Milan, Italy; linda.borgonovo@ospedaleniguarda.it (L.B.); marta.piantanida@ospedaleniguarda.it (M.P.); 5 Department of Clinical and Experimental Medicine, School and Operative Unit of Allergy and Clinical Immunology, University of Messina, 98122 Messina, Italy; federicabuta@gmail.com; 6 SC Medicina Generale 1, Fondazione IRCCS Policlinico San Matteo, 27100 Pavia, Italy; s.merli@smatteo.pv.it (S.M.); ca.rossi@smatteo.pv.it (C.M.R.); 7 Department of Internal Medicine and Medical Therapeutics, University of Pavia, 27100 Pavia, Italy; 8 Multidisciplinary Advanced Center Asthma, Food and Drug Allergy, IRCCS San Raffaele Scientific Institute, 21132 Milano, Italy; nannipieri.serena@hsr.it (S.N.); yacoub.monarita@hsr.it (M.-R.Y.); 9 Department of Public Health and Medical Science, University of Cagliari, 09124 Cagliari, Italy; giadasambugaro@gmail.com; 10 Department of Internal Medicine ASL Salerno, “Santa Maria della Speranza” Hospital, 84091 Salerno, Italy; patella@allergiasalerno3.it; 11 Faculty of Medicine and Surgery, University of Naples “Federico II”, 80138 Naples, Italy

**Keywords:** ICM, HSRs, ARs, IHSRs and NIHSRs, STs, intradermal test (IDT), Patch Tests (PTs), CR, drug provocation tests (DPTs), premedication, non-ionic low-osmolality contrast media, diagnosis and management

## Abstract

**Background/Objectives**: Hypersensitivity reactions (HSRs) to iodinated contrast media (ICM), both immediate and non-immediate, pose clinical challenges despite using low-osmolality agents. This review aims to summarize current diagnostic approaches, cross-reactivity patterns, and the debated role of premedication. **Methods**: A narrative review was conducted using PubMed (2014–2024), selecting studies on ICM-related HSRs, focusing on skin and in vitro testing, drug provocation tests (DPTs), cross-reactivity, and premedication. **Results**: Skin tests show limited sensitivity, especially for non-immediate reactions. Cross-reactivity among ICMs is common but unpredictable. DPTs are the diagnostic gold standard but lack standardized protocols. Premedication is frequently used, though its efficacy remains uncertain. **Conclusions**: The management of ICM hypersensitivity is limited by diagnostic gaps and insufficient evidence on premedication. Standardized protocols and prospective studies are needed to improve patient safety and guide clinical decisions.

## 1. Introduction

The introduction of iodinated contrast media (ICM) into clinical practice dates to the early 1920s. However, due to the poor radiopacity and toxicity of the compounds used, their use was limited until the introduction of new formulations in 1950, which provided better resolution and reduced toxicity. A further reduction in the toxicity of these compounds and, consequently, in the frequency of ARs occurred in 1970, with the production of non-ionic and hyposmolar ICM.

Nowadays, more than 75 million doses of ICM are administered annually. Consequentially the reported adverse reactions (ARs) to ICM are increased. The mechanism of ARs may be toxic (type A reactions) or immunological (type B reactions). HSRs are clinically distinguished by the time of symptoms’ appearance in immediate (IHSRs) and non-immediate (NIHSRs). The role of allergists is mandatory in the diagnosis and management of ICM HSRs, due to the importance of these drugs in radiological investigations [1]. The purpose of this narrative review is to take stock of what is in the literature on the recognition and diagnosis of allergic reactions to ICM with particular attention to some unmet needs, such as the validity and reliability of skin prick tests (STs), definition of cross-reactivity (CR) among various molecules, and role of drug provocation tests (DPTs) with evaluation of schemes proposed in the literature, as well as the debated role of premedication. This review aims to provide clinicians with an updated and structured synthesis of the available evidence on ICM hypersensitivity reactions, including both immediate and non-immediate forms. By addressing the current gaps in knowledge and clinical practice, such as the interpretation of diagnostic tests, patterns of cross-reactivity, and the debated role of premedication, we seek to offer practical insights and foster a more standardized approach to patient care.

## 2. Methods

We conducted a narrative review using the PubMed database to identify relevant literature published over the last 10 years (January 2014 to December 2024), focusing on hypersensitivity reactions to ICM. The search strategy included combinations of keywords such as “iodinated contrast media”, “hypersensitivity”, “skin testing”, “cross-reactivity”, “drug provocation test”, and “premedication”.

We included peer-reviewed publications in English reporting original data, clinical guidelines, consensus documents, systematic reviews, and relevant narrative reviews. Studies were selected based on clinical relevance, methodological rigor, and their contribution to diagnostic or therapeutic management. Particular attention was paid to multicenter studies and recent evidence from national or international expert panels.

The inclusion criteria were as follows: (1) studies involving adult or pediatric patients with suspected or confirmed hypersensitivity to ICM; (2) articles presenting clinical or laboratory-based diagnostic approaches (e.g., skin tests, in vitro tests, provocation testing); and (3) studies addressing treatment strategies or preventive approaches, including premedication protocols.

The exclusion criteria included the following: (1) non-English language publications; (2) preclinical or animal studies; (3) conference abstracts without full-text availability; and (4) papers lacking a clear focus on hypersensitivity reactions to ICM.

As this is a narrative review, the goal was not to exhaustively include all available studies but rather to select and discuss those with the highest clinical relevance and methodological rigor to support evidence-based allergological evaluation of ICM reactions.

The discussion was organized into six thematic areas to reflect the key clinical and scientific challenges encountered in the recognition, diagnosis, and management of hypersensitivity reactions to ICM.

## 3. Discussion

### 3.1. Pharmacological and Pathogenetic Correlations in ICM HSRs

An ideal contrast agent provides opacification without altering physiology or causing toxicity. Since 1929, iodine, which can increase X-ray absorption, has been used as a contrast agent. ICM features benzene rings with iodine and side chains that determine their physicochemical properties [2]. They are classified based on solubility and electrolytic dissociation into ionic and non-ionic [3,4]; the concentration of iodine, viscosity, and osmolarity influencing radiopacity; and the risk of ARs. The highest risk of ARs occurs with hypertonic high-osmolarity contrast media [5,6]. Nowadays, the most used and administered ICMs are the hydrosoluble, non-ionic, low-osmolarity ones. Indeed, they show good diagnostic performance and high tolerability. Lateral chains influence chemical–physical and pharmacological properties [7]. Several classifications have attempted to categorize ICM into distinct groups based on molecular characteristics (Table 1), but there is still no general agreement [8]. It is particularly relevant for clarifying the risk of CR between ICM, as explained in the specific subsection.

**Table 1:** Classification of non-ionic ICM based on sidechain. Adapted from Vega et al. [8].

Lerondeau et al. [9]	Schrijvers et al. [10]	Srisuwatchari et al. [11]	Seo et al. [12]
Group A: Iohexol, iomeprol, ioversol, iodixanol, and iopamidol [sharing 2 identical N-(2,3-dihydroxypropyl) carbamoyl sidechains]	Group A: Iohexol, iomeprol, ioversol, iodixanol, and iopromide [all sharing a N-(2,3-dihydroxypropyl) carbamoyl sidechain]	Group A: Iohexol, iomeprol, ioversol, iodixanol, iopromide, and iopamidol [with at least 1 N-(2,3-dihydroxypropyl) carbamoyl group]	Iohexol, iomeprol, ioversol, iodixanol, and iopromide [share a benzyl sidechain with an N-(2,3-dihydroxypropyl) carbamoyl group]. Iopromide and iobitridol have an N-(2,3-dihydroxypropyl)-N-methyl-carbamoyl group.
Group B: Iobitridol	Group B: Iobitridol and iopamidol	Group B: Iobitridol	Iopamidol does not possess either a N-(2,3-dihydroxypropyl) carbamoyl group or N-(2,3-dihydroxypropyl)-N-methyl-carbamoyl group.

As previously mentioned, ARs to ICM are distinguished into two main categories: toxic (type A) and HSRs (type B). The former is predictable and dose-dependent, often related to the physicochemical properties of the contrast agent, such as viscosity and osmolality. On the other hand, the latter are not dose-dependent and involve the release of mediators such as histamine, serotonin, and leukotrienes, which cause unpredictable clinical manifestations. These immediate and delayed reactions are related to pharmacological characteristics, such as osmolality, molecular structure, and excipient composition. Reduced osmolality and ionic neutrality minimize the activation of biological mediators, such as histamine, by mast cells and basophils. IHSRs are mediated by the direct or indirect activation of effector cells. Mast cell degranulation can occur through IgE-mediated or non-specific pathways. Only a minority of immediate reactions are IgE-mediated, as demonstrated by positive STs and elevated serum tryptase levels [13]. Concerning alternative responses, among the mechanisms considered to underlie immediate non-allergic reactions, ICM has a direct effect on cellular membranes. Indeed, ICM can act like a hapten, meaning it can trigger reactions even after the first exposure. The chance of a reaction increases as the osmolality of ICM rises. Additionally, they can independently induce the synthesis of bradykinin and activate the complement cascade or mast cell receptors, like Mas-Related G Protein-coupled Receptor X2 (MRGPRX2) [1,13,14]. NIHSRs primarily involve T lymphocytes and manifest with skin infiltration of CD4+ and CD8+ cells. The chemical structure of ICM has a significant influence on its ability to interact with plasma proteins and cell membranes. Studies show that non-ionic dimeric ICM can induce NIHSRs more frequently than monomeric ones. At the same time, ICM, with uniformly distributed hydrophilic side chains, reduces binding with proteins and cellular activation, thereby mitigating the risk of ARs. This underscores the importance of low-osmolality formulations in improving the tolerability and safety of ICM [15,16].

### 3.2. Epidemiology, Clinical Manifestations, and Risk Factors of HSRs to ICM

ARs to ICM occur in about 5% (mild), 0.022% (moderate), and 0.0025% (severe) of cases of reactions. Estimates of mortality are not reliable. Comparing the incidence between ARs related to ionic and non-ionic ICM, the former showed a four-fold higher risk compared to non-ionic ones [7]. HSRs to ICM affect around 0.5–3% of patients [17] and can happen right after administration or later. IHSRs occur within the first hour. Conversely, NIHSRs can take more than an hour to several days to appear [18]; their prevalence has been increasing during the last few decades and, currently, they are the most represented.

The clinical presentation of IHSRs varies from erythema/urticaria to anaphylactic reactions with severe consequences, such as cardiac arrest. Kounis syndrome is also described [19]. If more than one organ is affected, the probability of an IgE-mediated reaction rises, and cardiovascular signs are highly associated with allergy, especially when cutaneous or respiratory signs are present [20]. NIHSRs usually occur within three days of exposure and tend to resolve in one to seven days from the onset [21]. They generally affect 2–5% of patients who receive ICM [22]. Maculopapular rash and delayed urticaria are the most frequent manifestations [1]. Severe cutaneous adverse reactions (SCARs) are a heterogeneous group of delayed reactions associated with the administration of ICM. Stevens–Johnson syndrome (SJS), a severe mucocutaneous reaction characterized by widespread epidermal detachment, mucosal involvement, and systemic symptoms, such as fever, eosinophilia, or elevated liver enzymes, and other severe conditions, like acute generalized pustulosis (AGEP) and drug reaction with systemic symptoms and eosinophilia (DRESS), are described. Even if rare, the latter can also manifest after a few weeks from the exposure [23]. Symptoms and signs, such as fever, mucosal involvement, skin erosions or bullous lesions, or laboratory abnormalities (e.g., eosinophilia or elevated transaminases) are red flags that should worry physicians [24].

Given the ever-increasing number of procedures performed each year, it is paramount to understand the risk factors for HSRs to ICM, improve the safety of procedures, and avoid subjecting low-risk patients to unnecessary allergological investigations and premedication. A recent retrospective Italian study identified female gender and an age under 65 years as risk factors, along with cardiovascular disease, previous adverse drug reactions, and allergy to inhalants [25]. The Italian Guidelines identify medium-risk patients as those with uncontrolled bronchial asthma, ongoing urticaria and angioedema, recurrent angioedema, idiopathic anaphylaxis, and mastocytosis. High-risk patients are defined as those with a history of previous allergic reaction to ICM [26].

### 3.3. In Vivo and In Vitro Diagnosis of ICM HSRs

Diagnosing HSRs to ICM requires detailed medical history, STs, and, in some cases, in vitro assays. This approach is crucial for understanding reaction mechanisms and optimizing patient care and management. STs and in vitro tests should be performed between 6 weeks and 6 months after the reaction to improve sensitivity [27]. The skin prick test (SPT) and intradermal test (IDT) are first-line diagnostic tools. The SPT is useful in IgE-mediated reactions, with highly variable sensitivity that increases with the severity of the reaction. The SPT is performed undiluted with a broad panel of ICM, including those identified as potential culprits [16,26,27]. The IDT is important for diagnosing both IHSRs and NIHSRs; the evaluation is performed at 20 min and between 48 and 72 h after administration. The IDT should only be performed if the SPT has negative results, using dilutions from 1:1000 for severe cases to 1:10 for mild to moderate cases [27]. The sensitivity and specificity of STs vary based on different methods and studies examined. In IHSRs, sensitivity is approximately 20%, and specificity ranges from 96% to 100%. The negative predictive value (NPV) was 94.2% in IHSRs; however, it is difficult to estimate the positive predictive value (PPV) due to the paucity of data. The severity of the reaction also influences sensitivity: the more severe the reaction, the greater the sensitivity [4,28]. A recent review underlined that an undiluted IDT shows higher positivity compared to diluted IDT, with a significant increase in the right reactions for the culprit ICM, without causing irritating reactions. Therefore, it was recommended that the tests be started with diluted tests and, in the case of negative results with 1:10 dilution, that undiluted IDTs be proceeded with to improve sensitivity [29]. STs, in particular, delayed reading of IDTs and Patch Tests (PTs) with undiluted ICM are used in cases of suspicion of NIHSRs. They are evaluated at 48 h and between 96 and 120 h after administration [30]. One study reported an overall test positivity rate of 26%: respectively, 7% for SPTs, 22% for delayed reading of IDTs, and 16% for PTs [31]. Performing STs within six months of the reaction improves the positivity rate [32]. For SCARs, it is recommended to start with PTs due to their safety, despite being less sensitive than IDTs [27]. Indeed, even though in vivo tests are no longer considered a contraindication in the case of SCARs, especially if performed 6 months after complete resolution, the aim of STs should be to find safe alternatives rather than the culprit agent [24]. In addition to in vivo tests, in vitro assays are available for complex and severe reactions where STs cannot be performed. With a weak recommendation, the basophil activation test (BAT) is useful in IHSRs when the risk of a DPT is high [33]. For NIHSRs, the lymphocyte transformation test (LTT) has a sensitivity ranging from 13% to 75%, but it is mainly performed for research purposes due to its complexity [34].

### 3.4. Risk of Cross-Reactivity (CR) Between ICM

CR denotes the reactivity of a patient to multiple ICMs, usually due to chemical structure similarities within the same molecular class. This phenomenon complicates the prediction of safe alternatives in patients with allergies to ICM and is still partly elusive [27]. The prevalence of CR among ICM, as assessed by STs, varies widely across studies, ranging from 20 to higher than 75% and is related to the underlying pathophysiologic mechanism being significantly different from IHSRs and NIHSRs [32,35,36]. More precisely, IHSRs seem to be characterized by a lower spectrum of skin reactivity, which also includes mono-reactivity. This observation is probably related to the fact that IgE recognition in immediate reactions depends on high conformational (3D) affinity contact.

**Figure 1:**
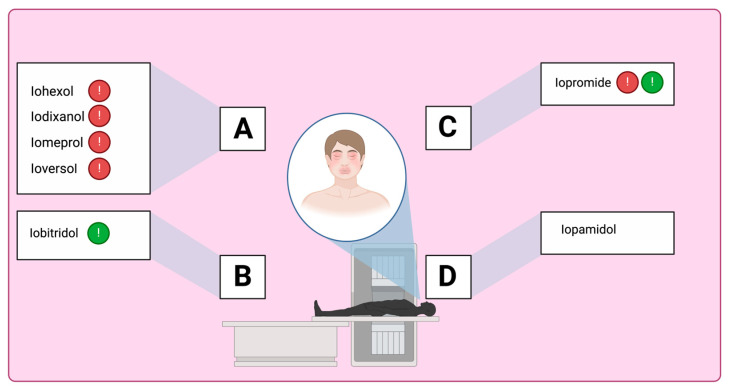
Alternative ICM classification based on a 2D structure focused on the carbamoyl side chain. ICM in Groups A and Group B is distinguished by different colors (red and green) based on the CR. Iopromide, in Group C, may cross-react with both Group A and Group B, while Iopamidol, in Group D, does not cross-react with any of the three. Adapted from Vega et al. [8].

**Figure 2:**
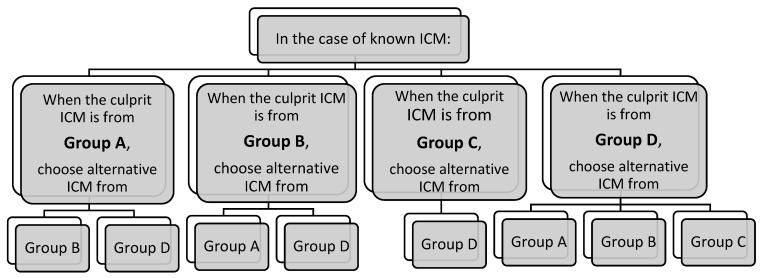
Empirically, a scheme for choosing an alternative ICM in the case of a known ICM. Adapted from Vega et al. [8].

**Figure 3:**
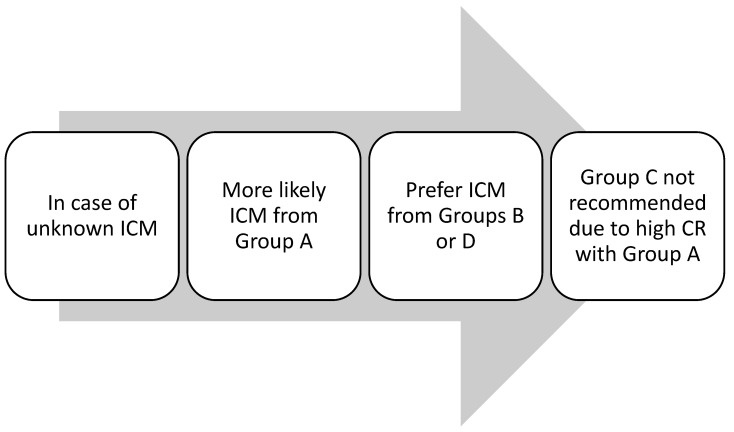
Empirically, a scheme for choosing an alternative ICM in the case of an unknown ICM. Adapted from Vega et al. [8].

In contrast, T cell receptor recognition in NIHSRs allows lower-affinity interaction with a wider array of linear epitopes. A chemical (2D) classification based on the presence of aliphatic moieties on the benzene ring identifies three to eight classes to explain STs’ reactivity. According to this classification, common associations are iohexol–ioversol, iohexol–iomeprol, iomeprol–ioverson, and iohexol–iodixanol for immediate reactions, and delayed iohexol–ioversol and iomeprol–ioversol for NIHSRs [37]. According to a recent meta-analysis including 7000 patients from six retrospective nonrandomized studies with a moderate risk of bias, a change in ICM is associated with a reduction of 60% in the immediate reaction rate in patients with prior HSRs to low-osmolality ICM [38]. In a study assessing the outcome of re-exposures after STs as the primary endpoint, sharing the same side chain was a factor associated with recurrence (20.7% vs. 11.5%, *p* = 0.003) in patients with severe reactions [12]. However, this classification does not explain some rarer patterns of ST reactivity. Vega et al. proposed an alternative classification based on a 2D structure, focusing on the carbamoyl side chain (Table 2 and Figure 1). They postulated that CR could be correlated with the carbamoyl side chain. An empirical strategy for choosing an alternative ICM based on this classification was suggested (Figure 2 and Figure 3) [8]. More recently, a conformational (3D) classification has been proposed. Accordingly, an uncommon association, such as that between ioxitalamate and amidotrizoate, has been found [37].

### 3.5. Protocols of DPTs

DPTs consist of the controlled administration of an ICM under medical supervision to observe ARs. DPTs are considered the gold standard for diagnosing IHSRs and non-severe NIHSRs when STs are inconclusive or negative. Still, they are rarely performed with the culprit agent for safety reasons. The primary application for DPTs is to verify tolerance to an alternative skin-test-negative ICM, as the NPV of STs is not well defined for reactions that occurred more than 6 months ago [28,39,40,41,42,43]. However, the use of DPTs in ICM hypersensitivity is debated [41,44] due to the following reasons:

1. The potential risks of ARs, either IHSRs or NIGHSRs, but also reactions due to toxicity, such as kidney injury, thyrotoxic crisis, and lactic acidosis. The assessment of serum creatinine and calculation of the estimated glomerular filtration rate before DPTs, as well as the monitoring of renal function, should be performed. Contraindications to perform DPTs are pregnant and breastfeeding women, patients with renal failure or patients taking nephrotoxic drugs, hyperthyroidism, and radioactive iodine therapy.
2. The lack of standardization: There is no consensus on the optimal dose of ICM for DPTs, as various doses have been used. For IHRs, challenge doses range from 10 mL to 120 mL, with the NPV of STs compared to DPTs varying between 37.5% and 100%. For NIHRs, doses between 10 mL and 100 mL have been used, showing a higher NPV of 66.6% to 100%. Concerning administration rates, some study protocols use increasing intravenous doses, reaching a total administration time of 4 h; others use a rapid full dose, administering 100 mL of ICM in 12 min.

The application of DPTs should be carefully considered on a case-by-case basis. Indeed, they should be performed only in well-equipped centers and by well-trained personnel.

In summary, DPTs should be considered in selected patients, particularly when skin tests are negative or inconclusive and re-exposure to ICM is clinically indicated. However, the risks associated with these procedures and the lack of standardized protocols highlight the need for further prospective research to validate their role in routine allergological practice.

### 3.6. Approach to Premedication

The approach to preventing new HSRs depends on the severity of the previous reaction. The risk–benefit ratio should be evaluated in patients at high risk of experiencing severe allergic reactions, particularly those with a history of such responses [45]. To prevent HSRs in high-risk patients, several premedication strategies have been proposed, mostly involving a combination of steroids and/or antihistamines; standard premedication regimens are usually administered orally, 12 to 13 h before the examination, or intravenously, beginning five hours before [46]. Greenberger et al. showed that corticosteroid and antihistamine preparation regimens before conventional high-osmolality ICM administration in high-risk patients led to a decrease in recurrence reaction by up to 11% [47]; however, to date, the use of low-osmolality ICM has become universal, primarily due to its significantly lower incidence of acute reactions. Therefore, the need for premedication in this context is less clearly defined, and there is insufficient evidence supporting the effectiveness of premedication in patients with a history of moderate or severe reactions. Some experts believe that premedication in high-risk patients reduces the rate of HSRs [5,48]. Kim et al. reported that premedication was effective. In a group of 11 patients with prior severe HSRs, only one premedicated patient experienced severe HSR when the same contrast agent was used again [49]. Many believe that steroids play a key role in both the prevention and treatment of moderate to severe HSRs. Some studies have shown that steroids may have a protective effect [48,50], while other studies have documented breakthrough reactions after using low-osmolar ICM, even with premedication using steroids [50,51]. There is no consensus on the premedication regimen, which varies depending on the hospital. As shown in Table 3, some examples from the literature are listed. The major limitations are that most of these studies are retrospective and lack solid conclusions on premedication efficacy; cohort studies and randomized controlled trials would be preferable and beneficial to assess the efficacy and role of premedication in managing patients with previous HSRs to ICM [52,53]. Overall, although premedication is commonly used in clinical practice for high-risk patients, the current evidence is largely derived from retrospective data. Its effectiveness remains uncertain, especially in patients with a history of severe HSRs. Prospective, controlled studies are required to define its preventive value better and to identify patients who are most likely to benefit from such interventions.

**Table 3:** Examples of premedication protocols. Adapted from Amiri [53].

Study	Premedication Scheme
Lee et al. [54]	For patients with a mild index reaction: 4 mg of intravenous (IV) chlorpheniramine 30 min before ICM administration.
	For patients with a moderate index reaction: 40 mg of IV methylprednisolone and 4 mg of IV chlorpheniramine 1 h before ICM administration.
	For patients with a severe index reaction: 40 mg of IV methylprednisolone 4 h and 1 h before and 4 mg of IV chlorpheniramine 1 h before ICM administration via the IV catheter inserted for ICM injection.
Park et al. [55]	No predefined premedication regimen but decided by physician in charge; histamines and steroids administered 0.5–1 h before exposure to ICM.
Mervak et al. [48]	Doses of 50 mg of prednisone administered 13 and 7 h and 1 h before CT (total, 150 mg prednisone), and 50 mg of diphenhydramine administered 1 h before CT.
Jung et al. [56]	No predefined premedication regimen but decided by physician in charge; histamines and steroids (usually 40 mg of methylprednisolone IV and 4 mg of chlorpheniramine and/or 20 mg of famotidine) administered 0.5–1 h before exposure to ICM.
Kolbe et al. [57]	Diphenhydramine alone (25–50 mg orally or intravenously 1 h prior to imaging); Corticosteroid alone (two doses of methylprednisolone 32 mg orally administered 12 and 2 h prior to imaging); Combined corticosteroid plus diphenhydramine.
Jha et al. [58]	IV corticosteroid (methylprednisone 125 mg, hydrocortisone 100 mg, or dexamethasone 16 mg), IV H2-blocker (famotidine 50 mg), and IV antihistamine (diphenhydramine 25 mg).
Bae Y et al. [59]	In case of mild reaction: chlorpheniramine, 4 mg alone, IV or intramuscularly 1 h before ICM; In case of severe reactions: IV hydrocortisone, 200 mg, plus chlorpheniramine, 4 mg 1 h before ICM.
Hubbard et al. [60]	In case of known previous AEs to ICM: methylprednisolone 80 mg IV, cimetidine 300 mg IV, prochlorperazine 10 mg orally, and montelukast 10 mg orally.

### 3.7. Unmet Needs, Evidence Gaps, and Future Directions

Despite growing attention toward hypersensitivity reactions to ICM, several unmet needs and limitations in the current evidence persist. First, the sensitivity and specificity of skin tests remain suboptimal and highly variable across centers, largely due to differences in methodology, interpretation criteria, and timing of testing. There is an urgent need for standardized protocols and consensus-based recommendations to improve diagnostic accuracy and inter-center reproducibility.

Second, although chemical classifications have attempted to predict cross-reactivity among ICMs, the underlying mechanisms, especially in non-immediate reactions, are not fully understood. This makes the identification of safe alternative agents particularly challenging in polysensitized patients or those with severe index reactions.

Third, DPTs, while considered the diagnostic gold standard, are underutilized in clinical practice due to safety concerns, heterogeneous dosing protocols, and a lack of prospective validation. Similarly, although premedication is widely used in high-risk patients, its efficacy, especially in preventing severe hypersensitivity reactions, remains controversial. The current evidence supporting its use stems primarily from retrospective studies, with few prospective trials available.

Moreover, a critical appraisal of the existing literature reveals that much of the available data derives from retrospective analyses, small single-center cohorts, or expert consensus statements. Randomized controlled trials and large prospective multicenter studies are notably lacking, particularly for evaluating the predictive value of skin and in vitro tests, the true utility of premedication, and the long-term safety of re-exposure strategies.

Future efforts should prioritize harmonized diagnostic algorithms, international registries, and collaborative research initiatives aimed at generating high-quality, evidence-based guidance for clinicians managing patients with suspected ICM hypersensitivity. Strengthening the methodological rigor of future studies will be essential to bridge the gap between current knowledge and clinical practice.

## 4. Conclusions

ICM improved the value of radiological evaluations and rapidly became a fundamental tool in every medical branch. Many radiological examinations with ICM are performed every day, and despite the reduction in the frequency of HSRs, especially after the introduction of non-ionic molecules, ARs continue to occur, and we still witness serious, potentially fatal anaphylaxis episodes. ARs to ICM may have both an immune-mediated and non-immune-mediated pathogenesis, with various underlying mechanisms. Unfortunately, there is no international consensus on the management of ARs to ICM. General recommendations involve replacing the culprit ICM with another structurally different or considered premedication. The main limitations are as follows:

- Low skin test sensitivity due to heterogeneous pathogenetic mechanisms and the time interval between reaction and allergy evaluation, which is often performed more than 6 months later;
- Potential cross-reactivity among ICM, which can affect up to 50% of skin tests;
- Lack of consensus about the real efficacy and safety of premedication in preventing the recurrence of adverse events;
- Lack of consensus on DPTs, regarding timing, dose, and administration rate.

The goal of our narrative review is to underline the crucial role of allergists in managing HSRs to ICM. The hope for the future is to conduct multicenter and prospective studies to standardize the approach to HSRs in ICM.

## Figures and Tables

**Table 2:** Alternative ICM classification based on a 2D structure focused on the carbamoyl side chain, figured in Groups A and B; images for Groups C and D represent the molecular structure of ICM iopromide and iopamidol. Adapted from Vega et al. [8].

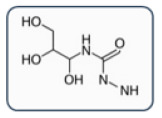	Group A: N-(2,3-dihydroxypropyl) carbamoyl side chains Iohexol Iodixanol Iomeprol Ioversol
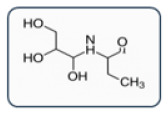	Group B: N-(2,3-dihydroxypropyl)-N-methyl carbamoyl side chains Iobitridol
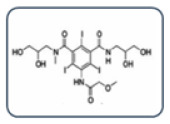	Group C: N-(2,3-dihydroxypropyl)-carbamoyl AND N(2,3-dihydroxypropyl)-N-methyl-carbamoyl side chains Iopromide
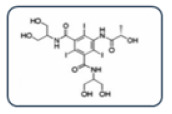	Group D: 1-N,3-N-bis(1,3 dihydroxypropan-2-yl) side chains Iopamidol

## Data Availability

No new data were created.

## Abbreviations

The following abbreviations are used in this manuscript:

ICM	Iodinated contrast media
ARs	Adverse reactions
HSRs	Hypersensitivity reactions
ST	Skin test
CR	Cross-reactivity
DTP	Drug provocation test
IHSRs	Immediate HSRs
NIHSRs	Non-immediate HSRs
MRGPRX2	Mas-Related G Protein-coupled Receptor X2
SCARs	Severe cutaneous adverse reactions
SJS	Stevens–Johnson syndrome
AGEP	Acute generalized pustulosis
DRESS	Drug reaction with systemic symptoms and eosinophilia
SPTs	Skin prick tests
IDTs	Intradermal tests
PTs	Patch Tests
BAT	Basophil activation test
LTT	Lymphocyte transformation test
NPV	Negative predictive value
PPV	Positive predictive value
IV	Intravenous
